# The heterogeneous landscape of ALK negative ALCL

**DOI:** 10.18632/oncotarget.14503

**Published:** 2017-01-04

**Authors:** Elisabetta Mereu, Elisa Pellegrino, Irene Scarfò, Giorgio Inghirami, Roberto Piva

**Affiliations:** ^1^ Department of Molecular Biotechnology and Health Sciences, Center for Experimental Research and Medical Studies, University of Torino, Torino, Italy; ^2^ Massachusetts General Hospital Cancer Center, Harvard Medical School, Boston, MA, USA; ^3^ Department of Pathology and Laboratory Medicine, Weill Cornell Medical College, New York, NY, USA

**Keywords:** anaplasticl large cell lymphoma, molecular classification, therapy, ALK negative

## Abstract

Anaplastic Large Cell Lymphoma (ALCL) is a clinical and biological heterogeneous disease including systemic ALK positive and ALK negative entities. Whereas ALK positive ALCLs are molecularly characterized and readily diagnosed, specific immunophenotypic or genetic features to define ALK negative ALCL are missing, and their distinction from other T-cell non-Hodgkin lymphomas (T-NHLs) can be controversial. In recent years, great advances have been made in dissecting the heterogeneity of ALK negative ALCLs and in providing new diagnostic and treatment options for these patients. A new revision of the World Health Organization (WHO) classification promoted ALK negative ALCL to a definite entity that includes cytogenetic subsets with prognostic implications. However, a further understanding of the genetic landscape of ALK negative ALCL is required to dictate more effective therapeutic strategies specifically tailored for each subgroup of patients.

## INTRODUCTION

Systemic Anaplastic Large Cell Lymphomas (ALCLs) refer to a group of malignancies of mature T lymphocytes characterized by large lymphoid cells (“hallmark cells”) and strong expression of CD30 [1]. The CD30 antigen has historically been instrumental in defining ALCL as a distinct category; however, its expression is not restricted to this pathology. CD30 is also found in activated non-neoplastic lymphoid cells [2, 3], in a subset of Peripheral T-cell Lymphoma - Not Otherwise Specified (PCTL-NOS) [4], in Hodgkin's lymphoma (HL) [1], and solid neoplasms [5]. The discovery of recurrent chromosomal translocations involving the anaplastic lymphoma kinase (ALK) gene in approximately 50% of ALCL patients [6] led to the delineation of ALK positive and ALK negative as two distinct subtypes [7, 8]. Of note, ALK activation is necessary and sufficient for promoting ALCL tumorigenesis and its inhibition is key for the therapeutic treatment of ALK positive ALCL [9–14]. Therefore, ALK positive ALCL was identified as a distinct disease. Conversely, ALK negative ALCL was defined as a provisional entity, lacking distinctive features.

In recent years, great advances have been made in dissecting the heterogeneity of ALK negative ALCLs and in providing new diagnostic and treatment options for these patients [15–20]. Consequently, the new revision of the World Health Organization (WHO) classification has promoted ALK negative ALCL to a definite entity that includes distinct cytogenetic subsets with prognostic implications [21].

This review will focus on advances in understanding the biology and pathogenesis of ALK negative ALCL, evaluating the clinical relevance of these findings.

## ALCLS FEATURES

Systemic ALCL comprises approximately 3% of all adult NHLs and 10% to 20% of childhood lymphomas. Both subtypes are characterized by male predominance (60%). Most patients present advanced stage disease (III to IV stage) often with B symptoms. ALK positive ALCL mostly affects young patients (10-19 years), whereas ALK negative ALCL occurs in older patients (peak of incidence in the sixth decade of life) [22]. Systemic ALCL frequently presents as a nodal disease, however extranodal involvement is seen in approximately 20% of cases, especially in skin, soft tissues, liver, and bone marrow.

ALK positive ALCL displays a better outcome compared to the ALK negative, with a 5-year overall survival rate of about 80% in ALK positive and 50% in ALK negative [6]. Nevertheless, when ALCL patients are stratified according to age and/or stage ALK positive and ALK negative individuals result in similar prognosis [23, 24].

ALCLs are characterized by high morphological heterogeneity, ranging from small-cell neoplasms to cases where very large and anaplastic cells predominate. However, almost all cases share a common feature, which is the presence of so called ‘hallmark cells’ characterized by abundant cytoplasm and large horseshoe-shaped nuclei. Neoplastic cells grow cohesively in a sheet-like pattern and preferentially involve lymph node sinuses [25].

Irrespective of ALCL subtype, strong expression of CD30 can be detected on the cell membrane and Golgi region. ALK positive ALCL cases show more often positivity for EMA (epithelial membrane antigen; 83% *vs* 43%; *P* < 0.001). Cytotoxic protein expression (TIA1, granzyme B, or perforin) is slightly more pronounced in ALK positive compared to ALK negative, but the difference is not statistically significant. ALK negative is most frequently CD3 positive compared to ALK positive ALCL [22, 23].

Another peculiar feature of ALCLs is the significant repression of the T-cell expression program. Even though nearly all ALCL cases (74-90%) show clonal TCR gene rearrangements, both ALK positive and ALK negative ALCLs lack T-cell receptors (TCRs) and related signaling molecules such as CD3, ZAP70, LAT and SLP76 [26, 27]. Paradoxically, ALCL cells display morphology, migration efficiency, and cytoskeletal rearrangements consistent with those of activated T-cells. In ALK positive ALCLs, it has been demonstrated that oncogene-deregulated tyrosine kinase activity controls T cell identity by transcriptional regulation and epigenetic silencing of key signaling molecules [28, 29].

Very recently Hassler et al. provided insights into the pathogenesis of ALCL through genome-wide DNA methylation profiling. The study found that ALK positive and ALK negative ALCL share common DNA methylation changes for genes involved in T cell differentiation and immune response [30].

A growing amount of literature has reported a link between breast implant and ALK negative ALCL designated as Breast Implant-Associated ALCL (BIA-ALCL or I-ALCL) [21]. Neoplastic cells are characterized by anaplastic features such as cytotoxic T-cell phenotype, CD30 and EMA co-expression, and ALK negativity [31–33]. Two distinct clinicopathological subtypes have been identified according to tumor localization: *in situ* BIA-ALCL (anaplastic cells confined to the fibrous capsule) and infiltrative I-ALCL (pleomorphic cells infiltrating adjacent tissue). *In situ* BIA-ALCLs have an indolent clinical course and generally remain free of disease after implant removal. On the contrary, infiltrative BIA-ALCLs have a more aggressive clinical course that might require systemic chemotherapy [34]. Chronic inflammation, implant immunogenicity, and sub-clinical infections have been implicated as driving mechanisms of BIA-ALCL tumorigenesis [32].

## THERAPEUTIC OPTIONS

Optimal therapy for ALK negative ALCL patients has not yet been identified due to the rarity of the disease and the lack of randomized trials.

CHOP (Cyclophosphamide, doxorubicin, vincristine, and prednisone), or CHOP-like regimen, is currently the standard of care in the initial management of ALCL patients [22].

After induction chemotherapy with CHOP, ALK negative ALCL patients often receive a high-dose chemotherapy followed by consolidative autologous stem cell transplantation [19, 21, 23, 35]. The outcome of ALK negative ALCL is consistently worse using CHOP-like regimens than in ALK positive ALCL and no improved survival rate could be achieved using dose-intensive chemotherapies [36, 37]. The poor outcome of ALK negative patients likely reflects the clinical and genetic heterogeneity of the disease and suggests that more specific therapeutic strategies should be explored.

In the last decade there have been a limited number of trials evaluating novel therapies specific for systemic ALK negative ALCL. Among these, CD30-directed therapies with Brentuximab Vedotin (BV) received great attention and displayed promising results [38]. BV is composed of an anti-CD30 antibody conjugated to the anti-microtubule agent monomethyl auristatin E (MMAE). Based on the positive responses to BV in relapsed/refractory ALCL (ORR: 86%; CR: 57%), the drug was approved in 2012 for relapsed/refractory ALCL following one line of therapy [39]. A subsequent study including 32 ALCLs patients (6 ALK positive and 26 ALK negative) demonstrated that BV treatment in combination with CHOP or CHP (CHOP without vincristine) exhibits substantial antitumor activity with a manageable safety profile (ORR: 100%; CR: 88%) [40]. The use of BV in combination with chemotherapy as front-line treatment is now being investigated in the ECHELON-2 phase III trial (NCT01777152).

## TRANSCRIPTIONAL PROFILES

Gene expression profiling (GEP) is a recognized tool to identify differentially expressed genes between two or more groups. This analysis has been widely applied to identify novel diagnostic and prognostic biomarkers for the peripheral T-cell lymphoma patients’ stratification [41–44].

Thompson et al. first demonstrated the ability of GEP to correctly distinguish between ALK positive and ALK negative ALCL based on the differential expression of genes encoding signal transduction molecules (*SYK, LYN, CDC37*), transcription factors (including *HOXC6* and *HOXA3*), and cell cycle regulators (*CCND3* and *CDKN2D*) [45].

A subsequent study performed on 32 systemic ALCL samples and 5 cell lines, identified ALK, BCL6, PTPN12, CEBPB, and SERPINA1 as the most discriminating genes between ALK positive and ALK negative ALCL. Moreover, a molecular signature of ALK negative included the overexpression of CCR7, CNTFR, IL22, and IL21 genes [46]. However, these studies have analyzed a small number of patients and lacked of objective quality control criteria.

A GEP analysis of T-cell non-Hodgkin's lymphoma samples, including angioimmunoblastic lymphomas (AILT), ALK positive ALCL, ALK negative ALCL, PTCL-NOS and normal T-cells, identified a genomic classifier for the recognition of ALCL patients [16]. Specifically, a set of 14 genes was able to distinguish ALK negative ALCL from PTCL-NOS and AILT. This study showed that ALCL patients share a cluster of transcripts, which allow their stratification and distinction from other T-cell lymphomas, and suggests that all ALCL may have a common cell of origin.

Piccaluga et al. developed a GEP-based molecular classifier that improved classification and prognostication among ALK negative ALCL, AITL, and PTCL-NOS patients [47]. This classifier displayed very high accuracy both in frozen and FFPE samples, however its clinical application remains limited due to the large number of genes required for ALK negative distinction.

A meta-analysis of several expression data sets [16, 48–50] identified and validated a 3-gene model (*TNFRSF8*, *BATF3*, and *TMOD1*) able to separate ALK negative ALCL from PTCL-NOS with a 97% accuracy [18]. The application of RT-qPCR protocols to FFPE tissues allowed the translation of GEP studies to routine clinical settings and the correct stratification of T-NHL.

To explore boundaries between PTCL-NOS and ALK negative ALCL, Bisig et al. analyzed the immunophenotype of different T-NHL subtypes [51]. The study found a substantial overlap between CD30-positive PTCL-NOS and ALK negative ALCL signatures. Specifically, CD30-positive PTCL-NOS were significantly enriched in ALK-negative related genes. The authors introduced a new hypothesis stating a biological continuum across CD30 positive PTCLs in contrast with other studies that demonstrated that PTCL-NOS and ALK negative ALCL are separated entities [16, 18, 23, 41]. The discordant observations were probably due to the high heterogeneity of these pathologies, different criteria for the samples’ characterization and the relatively small number of patients.

A more recent GEP analysis revealed that ALK negative ALCL were enriched for MYC and IRF4 target gene signature in comparison with PTCL-NOS [44]. The same study pointed out other differences between ALCL subtypes, such as the overexpression of the PI3K pathway- in ALK negative cases and the overrepresentation of HIF1A, IL10 and HRAS/KRAS-induced genes in the ALK positive patients. MYC inhibition has been demonstrated to be critical for ALCL survival and may represent a therapeutic target for ALCL therapy [52, 53].

ALK negative ALCL characterization was also improved by microRNA expression profiling. MicroRNAs (miRNAs) are small non-coding RNA molecules that play a crucial role in regulating gene expression at post-transcriptional level in a sequence-specific manner [54, 55]. miRNAs can act as oncogenes or tumor suppressors according to their target mRNAs. Recent works demonstrated the diagnostic and prognostic value of miRNA profiling for ALK negative patients. Liu et al proposed an 11-miRNA signature including 4 upregulated (miR-210, miR-197, miR-191, and miR-512-3p) and 7 downregulated miRNA (miR-451, miR- 146a, miR-22, miR-455-3p, miR-455-5p, miR-143, and miR-494), which distinguished ALK negative patients from PTCL-NOS with a 90% probability [56]. Merkel et al highlighted miRNA signatures associated with ALCL subtypes. The study described miR-17-92 cluster and miR-155 highly expressed in ALK positive and ALK negative ALCL patients, respectively [57]. Accordingly, Spaccarotella et al demonstrated that miR 17-92 cluster promotes proliferation and survival of ALK-positive anaplastic large cell lymphoma [58]. Moreover, miR-155 silencing results in increased levels of cleaved caspase-3 and SOCS1, which leads to STAT3 signaling suppression and tumor growth reduction in murine models of ALK negative ALCL [59]. These data suggested that miR-155 could act as a tumor driver in ALK negative ALCL. However, mir-155 is consistently over-expressed in the majority of T-NHL samples, indicating that its levels could not be used as a marker for differential diagnosis [18]. Another study identified a five miRNAs signature able to discriminate PTCL-NOS from ALK negative ALCL with high accuracy. This signature was validated in FFPE samples and was suggested to be predictive for the distinction between CD30 positive PTCL-NOS and ALK negative ALCLs [60]. Small RNA sequencing was recently used to investigate the differential expression of miRNA between ALCL subgroups. Steinhilber et al. identified a 56-miRNA signature distinguishing ALK positive, ALK negative and normal T-cells. This signature shows overlapping results with 26 miRNA identified by Merkel et al. and Liu et al. [61].

GEP has had a clear impact on elucidating ALK negative ALCL biology, defining the borders with other PTCL subtypes and providing new genomic classifiers for the correct stratification of patients. However, applying gene-expression profiling analysis is currently impractical and not yet standardized in routine clinical settings. Alternative strategies should be considered to translate the knowledge gained from GEP studies to the clinical arena. A three gene classifier able to discerne ALK negative ALCL showed potential clinical utility [18]. Recently, Nanostring nCounter technology has been developed to quantify a high number of RNA transcripts derived from formalin-fixed paraffin-embedded tissues [62, 63]. This methodology provides results concordant to conventional GEP with high reproducibility [64]. It is expected that the application of RT-qPCR or Nanostring protocols to FFPE tissues in clinical settings will allow the development of precise molecular diagnostic tools able to reduce errors and ambiguity in the stratification of T-NHL.

## SOMATIC COPY NUMBER ALTERATIONS

Comparative genomic hybridization (CGH) and single nucleotide polymorphism (SNP) arrays have thoroughly portrayed the profile of chromosomal imbalances of ALCLs. One of the first CGH studies in ALCLs and PTCL-NOS identified recurrent chromosomal gains of 1q (1q41-qter) in 46%, and losses of 6q (6q21) and 13q (13q21-q22) in 31% and 23% ALK negative ALCL patients, respectively [65]. The authors demonstrated that, despite a considerable overlap between the genetic features of ALK negative ALCL and PTCL-NOS (such as loss of 6q and 13q), the profile of chromosomal imbalances segregate PTCL-NOS from ALK negative ALCL.

Salaverria et al. performed CGH analysis in a large series of ALK positive and ALK negative ALCL [66]. Chromosomal imbalances were detected in 58% of ALK positive and 65% of ALK negative ALCL. ALK positive ALCL cases displayed recurrent 17p and 17q24-qter gains and 4q13-q21, and 11q14 losses, gains of 1q and 6p21 were more frequently observed in ALK negative ALCL, whereas gains of chromosome 7 and 6q and 13q losses were seen in both types of ALCL tumors. The authors demonstrated that ALK positive and ALK negative ALCL harbor different genetic aberrations, confirming that they correspond to separated genetic entities.

More recently, the genomic profile of ALCL was analyzed with a different approach. Genome-wide DNA profiling of ALCL using high-density, single nucleotide polymorphism (SNP) arrays identified concomitant losses at 17p13 and at 6q21, encompassing the TP53 and PRDM1/BLIMP1, in up to one quarter of ALCL cases. Loss of TP53 and/or PRDM1 was present in 52% ALK negative ALCL, and in 29% of all ALCL cases. In particular, PRDM1 displayed a tumor suppressive role in the ALCL model [67]. BLIMP1 is a critical factor for B and T cell differentiation, and its onco-suppressive role has been documented in different models including diffuse large B cell lymphomas and natural killer cell lymphoma [68].

Copy number alteration studies have provided the landscape of chromosomal aberrations in ALCLs. However, these findings did not find their application in the routine clinical setting.

## MUTATIONS

Using classical DNA Sanger sequencing, PRF1 monoallelic germline mutations were frequently found in patients with childhood ALCL (27% of cases). Current opinion is that PRF1 mutations are not oncogenic per se but they could represent a predisposition factor for the disease by partially impairing the cytotoxic machinery [69, 70].

Next generation sequencing (NGS) technologies have emerged over the past decade providing new insights into the biology of ALCL. Different NGS based approaches ranging from amplicon-based (targeted), whole exome or whole genome sequencing were applied to identify new translocations and somatic mutations in ALK negative ALCL [17, 71, 72].

Whole exome sequencing was used to investigate the frequency of somatic mutation and associated to copy number variation analysis in ALK negative ALCL [71]. Among the plethora of mutations including PRDM1, TP53, TET2, FAS and STIM2 genes, JAK1 and STAT3 genes were the most recurrently mutated accounting for 18% of systemic ALK negative ALCL. The authors demonstrated that JAK1/STAT3 mutations lead to STAT3 activation and transformation. Interestingly, constitutive STAT3 phosphorylation was observed in a significant proportion of JAK1/STAT3 non-mutated cases, suggesting alternative mechanisms of pathway activation.

JAK/STAT3 signaling is frequently deregulated in hematopoietic and solid tumors [73]. In ALK positive ALCL, the oncogenic effect of ALK chimeras is mostly mediated by STAT3 [74–78]. The discovery of STAT3 activation in ALK negative ALCLs suggests that the STAT3-mediated oncogenic mechanism may be shared by all ALCLs, independently of ALK status. As a result of these findings Crescenzo et al. demonstrated that JAK/STAT3 inhibition impaired tumor growth in a preclinical ALK-negative ALCL-patient derived tumorgraft model, providing new potential therapeutic targets for ALK negative treatment [71]. Activating STAT3 mutations have been observed in other T cell and B cell disorders [79, 80], suggesting that STAT3 might be considered as a therapeutic target in several malignancies. Among JAK/STAT3 pathway inhibitors, Ruxolitinib displays promising results in different pathological models and has been approved by the FDA to treat myeloproliferative disorders [81]. The identification of ALCL patients that could benefit from this therapy can be promptly achieved by immunohistochemical staining for activated STAT3.

## CHROMOSOMAL TRANSLOCATIONS

Next generation sequencing performed on mate-paired libraries, identified numerous rearrangements in ALK negative ALCL. The first translocation described involves DUSP22-IRF4 locus on 6p25.3 and the FRA7H fragile site on 7q32.3 [17]. The presence of a DUSP22 rearrangement was associated with down-regulation of DUSP22 expression and upregulation of mir29A levels. DUSP22 is a dual-specificity phosphatase involved in JNK activation [82, 83], suppression of IL-6-induced STAT3 activation [84] and TCR signaling down-regulation in reactive T cells through ERK2 inactivation [85]. DUSP22 has a tumor suppressor function in B-cell lymphomas [86], T-lymphoblastic leukemias [87] and ALK positive ALCLs [88]. FRAH7H site contains a miRNA gene cluster that includes miR-29b. Accordingly, ALCL with 7q32.3 rearrangements show miR-29b over expression. The role of miR-29b is still controversial, however its up-regulation suggests a role as tumor promoter in ALCL, AML [89], bladder cancer [90], and breast cancer [91]. At present, the biological significance of DUSP22 rearrangements has still to be demonstrated.

With the same approach thirteen recurrent rearrangements were identified in PTCLs, five of these are p53-related genes, including TP53, TP63, CDKN2A, WWOX, and ANKRD11. The authors focused their attention on inv(3)(q26q28) that leads to the expression of a fusion transcript TBL1XR1/TP63 with structural homology to oncogenic deltaNp63, a p63 isoform lacking transactivation domain [72]. DeltaNp63 acts as a dominant negative by inhibiting the p53 pathway. Its oncogenic role has been demonstrated in several models including breast [92, 93], lung [94], and head and neck cancers [95]. TP63 rearrangements were exclusively found in PTCL-NOS (9.4%), ALK negative ALCL (12.5%), and primary cutaneous ALCL (10.5%). Of note, the large majority of TP63-positive PTCL-NOS show CD30 expression higher than 80%. Moreover, TP63 was associated to inferior overall survival [72].

Subsequent analyses were aimed to test DUSP22 and TP63 rearrangements as biomarkers for diagnosis and risk stratification of ALK negative ALCL patients. A multi-institutional study on 105 ALCL patients (32 ALK positive and 73 ALK negative) revealed that DUSP22 and TP63 rearrangements are present in 30% and 8% of ALK-negative ALCL patients, respectively. These rearrangements were mutually exclusive and specifically expressed in ALK negative ALCL. On morphologic grounds DUSP22-rearranged ALCL display significant differences from other ALK negative ALCL, typically showing sheet-like growth with doughnut cells and few large pleomorphic cells. Tumor cells are CD30 positive, ALK, TIA-1 and granzyme B negative. TP-63 rearranged cases show more heterogenic features with hallmark cells always present. However, the small number of TP63-rearranged cases limited the identification of general features [19, 96].

Patients with DUSP22 rearrangement had better outcomes, similar to ALK positive ALCL (five years overall survival: 90% DUSP22 and 85% ALK positive ALCL). Patients with TP63-rearrangements had overall survival rates significantly worse than those with ALK positive ALCL (five years overall survival: 17%). Therefore, DUSP22 and TP63 rearrangements have important prognostic relevance and may serve as predictive biomarkers [19].

**Figure 1:**
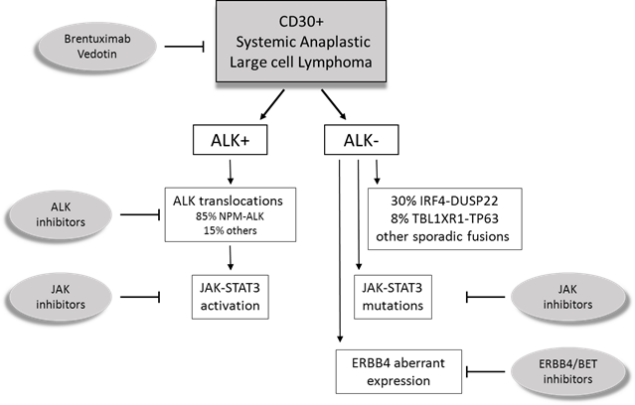
Schematic representation of systemic Anaplastic Large Cell Lymphomas (ALCL) ALK positive (ALK+) ALCL is a well-defined entity, characterized by ALK translocations. ALK negative (ALK-) ALCL were promoted to a definite entity that includes different cytogenetic subsets with prognostic and pharmacological significance.

The study by Crescenzo et al. further depicted the heterogeneity of ALK negative ALCL by the recognition of numerous sporadic fusion transcripts [71]. As a common feature, chimeric proteins recurrently involved tyrosine kinases (i.e. ROS1 or TYK2) triggering the activation of JAK/STAT3 pathway. Interestingly, these gene fusions were mutually exclusive with JAK1/STAT3 mutations, suggesting convergent pathogenetic mechanisms and therapeutic targets for ALK negative ALCL (Figure 1).

Whole genome sequencing analyses have shed light on genetic and biological heterogeneity among ALK negative ALCL, supporting the idea that this entity is composed by different subgroups. DUSP22 and TP63 translocations defined three different subgroups (DUPSP22-positive, TP63-positive and triple negative ALCL) with clear prognostic implications. The predictive value of these rearrangements can be successfully translated to routine clinical setting by performing fluorescence *in situ* hybridization assays, in order to deliver the most appropriated therapeutic protocol to ALK negative patients. Early autologous Stem Cell Transplantation (SCT) is indicated for ALK negative patients but not for ALK positive because of their favorable outcomes following chemotherapy. The discovery that DUSP22 positive patients show similar outcomes to ALK positive may play a role in the decision to employ early SCT [97].

ROS and TYK translocations assume clinical value in the light of their ability to activate JAK/STAT pathway which represent an attractive therapeutic target for ALK negative ALCL, as discussed above. Immunofluorescence analysis for routine clinical detection of these translocations in primary samples remains to be confirmed on a larger cohort of ALCL patients.

## ABERRANT TRANSCRIPTS

Using integrated bioinformatics approaches Scarfò et al. recently identified a novel diagnostic subclass of ALK negative ALCL coexpressing ERBB4 and COL29A1 and featuring a specific gene signature [20]. ERBB4 encodes for a member of the tyrosine kinase receptor superfamily which includes ERBB1 (EGFR) and ERBB2 (HER2), known to be deregulated in several solid tumors [98]. ERBB4 was found to be mutated and potentially oncogenic in several cancer types, such as melanoma and lung adenocarcinoma [99–103]. Moreover, it has been demonstrated that ERBB4 mediates acquired resistance to ERBB2 inhibitors in breast cancer cells [104, 105]. Very recently, Boddicker et al. described a novel translocation involving ERBB4 in one PTCL-NOS patient. This rearrangement contains IKZF2 gene (exons 1-2) fused with ERBB4 (exons 2-28) [106].

Scarfò et al. found ERBB4 expression in ~25% ALK negative ALCL, but not in PTCL-NOS nor in ALK positive ALCL. Interestingly, ERBB4 ectopic expression in ALK negative ALCL patients resulted from two different truncated transcripts: I20ΔERBB4 and I12ΔERBB4. The study suggests that ERBB4 aberrant expression is not due to a genomic alteration, rather it is driven by reactivation of normally dormant long terminal repeat elements (LTRs) located in ERBB4 introns [20]. Examples of ancient LTR promoters awakening have been previously reported in Hodgkin lymphoma (HL). Lamprecht and colleagues demonstrated that the activation of an endogenous LTR leads to the expression of colony-stimulating factor 1 receptor (CSF1R), which results oncogenic and correlates with a poor outcome of HL patients [107]. More recently, Babaian et al. reported the activation of the LOR1a LTR with consequent ectopic overexpression of IRF5 [108]. Notably, ERBB4 positive ALCL frequently displayed Hodgkin-like features, usually rare among conventional ALCL. Considering the shared awakening of ancient LTR promoters in HL and ERBB4 positive ALCL, it will be interesting to analyze ERBB4 expression in Hodgkin Lymphoma samples.

The study by Scarfò et al. indicated that ERBB4-truncated forms show oncogenic potentials. Nevertheless, the pharmacologic inhibition of ERBB4 only partially controls ALCL cell growth and disease progression in a preclinical model, indicating the need for combination therapies in relapsed or refractory ERBB4-positive ALCL patients.

The identification of a subset of ERBB4 expressing ALK negative ALCL confirms the commonly accepted hypothesis that ALK negative includes multiple subgroups driven by different aberrations. ERBB4 represents a new diagnostic marker and a potential therapeutic target for this novel subclass. Clinical diagnosis of ERBB4 positive patients can be established using droplet digital PCR analysis for ERBB4 detection and/or immunohistochemical staining for MMP9, a protein highly correlated with ERBB4 expression. The diagnostic value of this finding is reinforced by observing that ERBB4 expression is mutually exclusive with other rearrangements such as: TP63, DUSP22 and ROS or TYK translocations. However these data have to be confirmed in a larger panel of patients.

LTR de-repression raises the perspective of clinical use of epigenetic drugs in tumors driven by transposable elements (TE) and refractory to current standard therapeutic regimens. Histone methylation and acetylation are among the most relevant modifications that guide chromatin remodeling and epigenetic control [109]. Targeting genes that regulate these modifications can represent valid therapeutic strategies to repress TE-driven oncogenic transcription. Indeed, the use of demethylases (KDM) and bromodomains (BET) inhibitors to block aberrant transcription could be an attractive option [110]. In particular, numerous studies have highlighted BET inhibitors as a novel category of anti-cancer agents, with preclinical and clinical evidence both in solid and hematological malignancies [111–113].

## CONCLUSIONS

ALK negative ALCL is a genetically and biologically heterogeneous neoplasm previously considered a provisional entity because of the lack of specific biomarker. Many efforts have been made over the past few decades to identify precise, reproducible and clinically applicable biomarkers that have led to the recognition of ALK negative ALCL as a distinct clinicopathologic entity. The discovery of key driver mutations and therapeutic targets has been slowed down by the intrinsic molecular heterogeneity and the relative rarity of this disease. However, recent advances in next generation sequencing and bioinformatics approaches allowed the recognition of different subgroups of ALK negative ALCL with prognostic or pharmacological relevance. Integrating these findings with each other will be critical to understand the molecular heterogeneity of ALK negative ALCL and to select therapeutic strategies specifically tailored for each subgroup of ALK negative patients.
